# The complete chloroplast genome of *Myriophyllum spicatum* reveals a 4‐kb inversion and new insights regarding plastome evolution in Haloragaceae

**DOI:** 10.1002/ece3.6125

**Published:** 2020-03-04

**Authors:** Yi‐Ying Liao, Yu Liu, Xing Liu, Tian‐Feng Lü, Ruth Wambui Mbichi, Tao Wan, Fan Liu

**Affiliations:** ^1^ Key Laboratory of Southern Subtropical Plant Diversity Fairy Lake Botanical Garden Shenzhen China; ^2^ Laboratory of Plant Systematics and Evolutionary Biology College of Life Science Wuhan University Wuhan China; ^3^ Sino‐Africa Joint Research Centre Chinese Academy of Science Wuhan China; ^4^ Key Laboratory of Aquatic Botany and Watershed Ecology Wuhan Botanical Garden Chinese Academy of Sciences Wuhan China

**Keywords:** Haloragaceae, hydrophyte, inversion, *Myriophyllum spicatum*, structure variation

## Abstract

*Myriophyllum*, among the most species‐rich genera of aquatic angiosperms with ca. 68 species, is an extensively distributed hydrophyte lineage in the cosmopolitan family Haloragaceae. The chloroplast (cp) genome is useful in the study of genetic evolution, phylogenetic analysis, and molecular dating of controversial taxa. Here, we sequenced and assembled the whole chloroplast genome of *Myriophyllum spicatum* L. and compared it to other species in the order Saxifragales. The complete chloroplast genome sequence of *M. spicatum* is 158,858 bp long and displays a quadripartite structure with two inverted repeats (IR) separating the large single copy (LSC) region from the small single copy (SSC) region. Based on sequence identification and the phylogenetic analysis, a 4‐kb phylogenetically informative inversion between *trn*E‐*trn*C in *Myriophyllum* was determined, and we have placed this inversion on a lineage specific to *Myriophyllum* and its close relatives. The divergence time estimation suggested that the *trn*E‐*trn*C inversion possibly occurred between the upper Cretaceous (72.54 MYA) and middle Eocene (47.28 MYA) before the divergence of *Myriophyllum* from its most recent common ancestor. The unique 4‐kb inversion might be caused by an occurrence of nonrandom recombination associated with climate changes around the K‐Pg boundary, making it interesting for future evolutionary investigations.

## Graphical abstract

We sequenced the entire chloroplast genome of *Myriophyllum spicatum*, a species in Saxifragales. The chloroplast genome of *M. spicatum* is 158,858 bp long and consists of a quadripartite structure with two inverted repeats separating the large and small single copy regions. In addition, it contains a 4‐kb phylogenetically informative inversion between trnE‐trnC that is unique to at least two genera in Haloragaceae. This inversion may be useful as a barcode to identify species in this genus, many of which are invasive and difficult to identify using morphological characteristics.

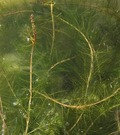

## 1 INTRODUCTION

Haloragaceae (Saxifragales) is a dicotyledonous, cosmopolitan family that includes eight genera and approximately 138 species (Moody & Les, 2007). Life forms vary widely in this family, which presents both terrestrial (small trees, shrubs, subshrubs, and annuals) and aquatic or semiaquatic genera (Moody & Les, 2010). *Myriophyllum* L. is a cosmopolitan aquatic angiosperm genus in Haloragaceae with ca. 68 species (as defined by APG II 2003). Some *Myriophyllum* species are highly invasive in several countries due to rapid asexual reproduction and strong competitiveness in aquatic systems (Moody & Les, 2010). In addition, reliable morphological identification of *Myriophyllum* is particularly difficult in the field when reproductive structures are lacking, as is common among many aquatic taxa (Cronk & Fennessy, 2001; Moody & Les, 2010; Sculthorpe, 1967). The genetic relationships also do not readily facilitate identification as previously published molecular phylogenies are lacking (Moody & Les, 2010). Commonly used markers for determining phylogenetic relationships include the nuclear‐encoded internal transcribed spacer (nrITS) and numerous chloroplast DNA markers (Moody & Les, 2007, 2010; Thum, Zuellig, Johnson, Moody, & Vossbrinck, 2011). Therefore, it is necessary to select more appropriate phylogenetically informative regions.

The sequencing of whole chloroplast genomes (cp genome), which are haploid and maternally inherited, have the potential to significantly advance our ability to resolve evolutionary relationships in complex plant lineages, such as *Myriophyllum* (Doorduin et al., 2011; Philippe & Roure, 2011). The plant cp genome is generally conserved in content and structure. It is usually composed of two copies of inverted repeats (IR) that separate a large single copy region (LSC) from a small single copy region (SSC). Highly conserved genes (100–120) have been retained in the cp genome, including those for photosynthesis, self‐reproduction, transcription of chloroplast expression‐related genes, and some unknown genes (Wicke, Schneeweiss, Depamphilis, Müller, & Quandt, 2011). Despite being much more conservative than the nuclear and mitochondrial genomes, the cp genome still varies in size, contraction and expansion of IRs, and structure (Daniell, Lin, Yu, & Chang, 2016). Moreover, many mutation events in the cp genome have been detected including indels, substitutions, and inversions (Chumley et al., 2006).

These evolutionary hotspots can provide useful information to elucidate the phylogenetic relationships of taxonomically unresolved plant taxa. Kim, Choi, and Jansen (2005) confirmed the Barnadesioideae as the most basal lineage in the Asteraceae by using a 22‐kb DNA inversion. The close relationship between the Poaceae and Joinvilleaceae was clarified by treating three DNA inversions composed of a nested set as a phylogenetic character (Doyle, Davis, Soreng, Garvin, & Anderson, 1992). Some variations in the cp genome, like gene loss and transfer, have been used to determine the evolutionary history of some plant species. For example, the extreme loss of *ndh* genes observed in *Najas flexilis* was used to illustrate a modified character associated with photosynthetic efficiency (Peredo, King, & Les, 2013).

**Figure 1:**
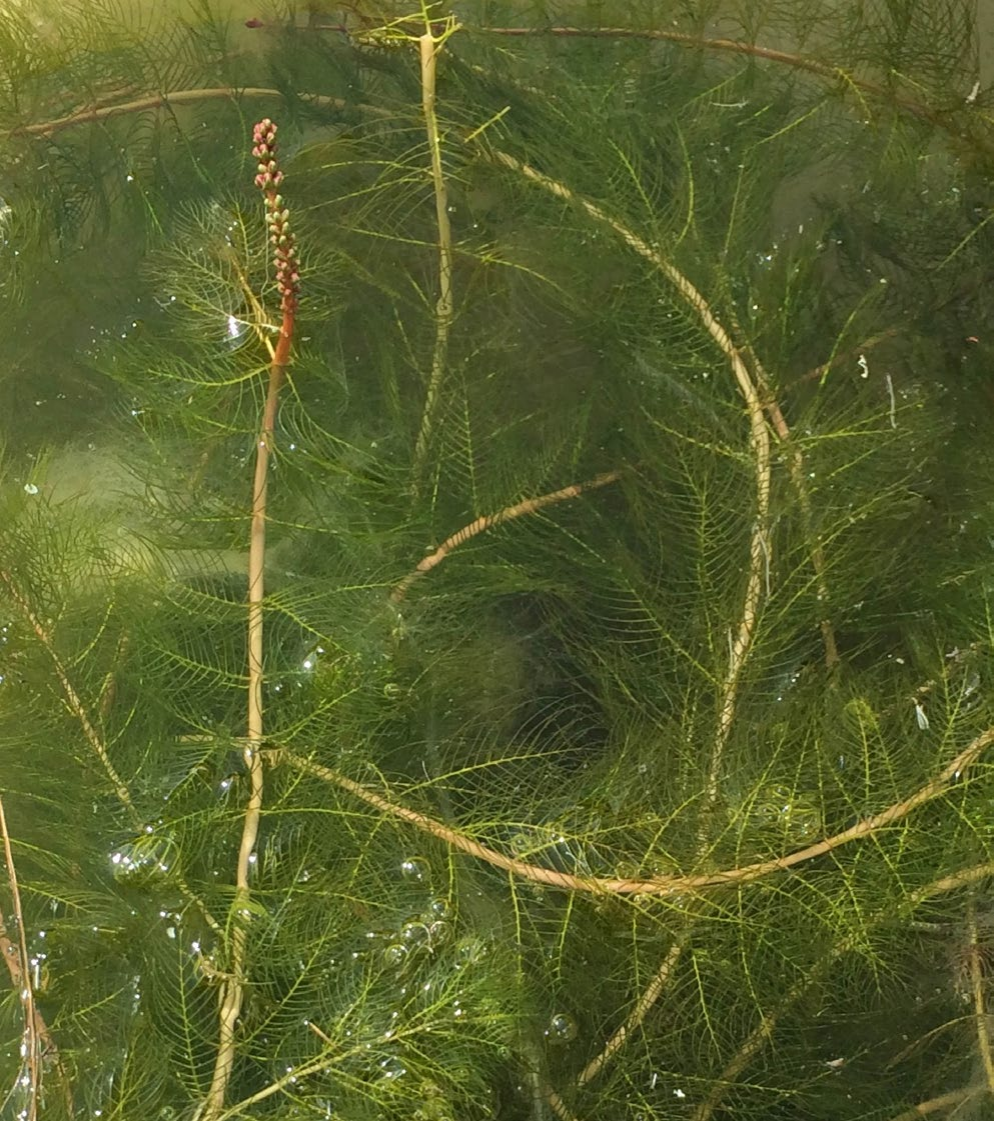
The *Myriophyllum spicatum* L. (Haloragaceae, *Myriophyllum*), a perennial submerged aquatic plant widely distributed in Europe, Asia, and north Africa

In this study, we sequenced the complete cp genome of *M. spicatum* (Figure 1). The cp genome was then compared with previously published cp genomes from related species, allowing the identification of a noteworthy inversion. Phylogenetic analyses were then performed on Saxifragales spp. to determine the point at which the inversion in the cp genome of *Myriophyllum* occurred. Finally, we evaluated the sequence divergence between *Myriophyllum* and other clades in Haloragaceae. We investigated potentially useful plastid regions for future molecular phylogenetic analyses in Saxifragales with observation on the variation of chloroplasts at different molecular markers (exon, intron, and intergenic regions). These data provide insight into the evolutionary history of this cosmopolitan family and, in the future, will facilitate the identification of *Myriophyllum* spp.

## 2 MATERIALS AND METHODS

### 2.1 Plant materials and DNA extraction

The taxa sampled in this study are shown in Table 1. All specimens were deposited in Wuhan Botanical Garden, Chinese Academy of Sciences in China. The total DNA of all samples were isolated from the fresh leaves according to the mCTAB method (Li, Wang, Yu, & Wang, 2013).

**Table 1:** Taxa used for cp DNA sequencing and PCR diagnosis of the inversion

No.	Family	Species	Locality	Used for
1	Haloragidaceae	*Gonocarpus micranthus* Thunb.	Shanwei, Guangdong, China	cp DNA sequencing
2	Haloragidaceae	*Myriophyllum alterniflorum* DC.	UK	PCR diagnosis
3	Haloragidaceae	*Myriophyllum aquaticum* (Vell.) Verdc.	Zhenjiang, Jiangsu, China	PCR diagnosis
4	Haloragidaceae	*Myriophyllum dicoccum* F. Muell.	Shanwei, Guangdong, China	PCR diagnosis
5	Haloragidaceae	*Myriophyllum heterophyllum* Michx.	USA	PCR diagnosis
6	Haloragidaceae	*Myriophyllum lophatum* Orchard	Australia	PCR diagnosis
7	Haloragidaceae	*Myriophyllum oguraense* Miki	Liangzi Lake, Ezhou, Hubei, China	PCR diagnosis
8	Haloragidaceae	*Myriophyllum quitense* Kunth	USA	PCR diagnosis
9	Haloragidaceae	*Myriophyllum sibiricum* Kom.	Ice land	PCR diagnosis
10	Haloragidaceae	*Myriophyllum spicatum* L.	Germany	PCR diagnosis
11	Haloragidaceae	*Myriophyllum tenellum* Bigelow	USA	PCR diagnosis
12	Haloragidaceae	*Myriophyllum ussuriense* Maxim.	Wuhan Botanical Gardon, Wuhan, Hubei, China	PCR diagnosis
13	Haloragidaceae	*Myriophyllum variifolium* Hook.f.	Australia	PCR diagnosis
14	Haloragidaceae	*Myriophyllum verrucosum* Lindl.	Australia	PCR diagnosis
15	Haloragidaceae	*Myriophyllum verticillatum* L.	Fuyuan, Heilongjiang, China	PCR diagnosis

### 2.2 Chloroplast genome sequencing, mapping, and annotation for *M. spicatum*

The whole cp genome of *M. spicatum* was sequenced. The DNA sequencing library of *M. spicatum* was prepared following the method described by Dong, Xu, Cheng, Lin, and Zhou (2013) and Dong, Xu, Cheng, and Zhou (2013), and fragments were amplified using universal primers. Specific primers were designed for regions, such as poly‐A tails, that were insufficiently amplified using the universal primers. The inverted repeat regions (IRs) of the cpDNA were not amplified separately; instead, primers were designed to amplify the regions spanning the junctions of LSC/IRA, LSC/IRB, SSC/IRA, and SSC/IRB. Using these primers, we covered the entire cp genome of *M. spicatum* with PCR products ranging in size from 500 bp to 5 kb. The overlapping regions of each pair of adjacent PCR fragments exceeded 150 bp. The standard PCR amplification reactions were performed at 94°C for 4 min followed by 35 cycles of 30s denaturation at 94°C, 30s annealing at 55°C, 1.5 min extension at 72°C, and a final extension of 72°C for 10 min. PCR products were electrophoresed on a 1.0% agarose gel and purified with gel extraction kit (Omega Bio‐Tek). The amplified DNA fragments were further sent to Majorbio Bio‐Pharm Technology Co. Ltd. (Shanghai, China) for Sanger sequencing in both the forward and reverse directions according to their standard protocols on an ABI 3730xl DNA Analyzer. All fragments were sequenced 2–10 times (6‐fold coverage of the *M. spicatum* cp genome on average). The chloroplast DNA sequences were manually assembled by using of the program Sequencher v4.1.4 (Gene Codes Corporation, USA). Since automated assembly methods cannot distinguish two IRs, we input the reads as two groups and obtained two large contigs, with each contig including one IR and its adjacent partial large and small single copy (LSC and SSC) regions. Then, the two large contigs were manually assembled into the complete circular genome sequence.

The cp genome of *M. spicatum* was annotated using the online program Dual Organellar Genome Annotator (DOGMA; Wyman, Jansen, & Boore, 2004). All tRNA genes were further verified by the corresponding structures predicted by tRNAscan‐SE 1.3.1 (Schattner, Brooks, & Lowe, 2005). The graphical map of the circular plastome was drawn by GenomeVx (Conant & Wolfe, 2008).

The frequency of codon usage in exon sequences of all protein‐coding genes of the cp genome of *M. spicatum* was calculated by using of MEGA 6 (Tamura, Stecher, Peterson, Filipski, & Kumar, 2013) and yn00 in PAML 4 (Yang, 2007). REPuter (Kurtz et al., 2001) was used to identify and locate forward, palindrome, reverse, and complement sequences that were ≥30 bp and had a sequence identity ≥90%. Simple sequence repeats (SSRs) were identified with MISA (http://pgrc.ipk-gatersleben.de/misa/; Thiel et al., 2003). Detection criteria were constrained to perfect repeat motifs of 1–6 bp and a minimum repeat number of 8, 4, 4, 3, 3, and 3, for mono‐, di‐, tri‐, tetra‐, penta‐, and hexa‐nucleotide repeats, respectively. Geneious v8.0.2 (http://www.Geneious.com; Kearse et al., 2012) was used to perform the mapping of the location and size of repeated elements and SSRs in the *M. spicatum* cp genome.

### 2.3 Comparative genomic analysis

To determine structural variation of the cp genome, the newly sequenced cp genome of *M. spicatum* was compared with the cp genome of four other Saxifragales species: *Liquidambar formosana* [KC588388], *Paeonia obovata* [KJ206533], *Penthorum chinense* [JX436155], and *Sedum sarmentosum* [JX427551]. Mauve software 2.3.1 was used to determine the structural variation (Darling, Mau, Blattner, & Perna, 2004), and the cp genome of *Nicotiana tabacum* [NC_001879] was used as a reference.

To identify the presence of large structural variation (>1 kb) within the *M. spicatum* plastome, breaks of synteny were searched among plastomes of *M. spicatum*, *L. formosana*, *P. obovata*, *P. chinense*, and *S. sarmentosum* as well as two outgroup taxa, *Vitis vinifera* [NC_007957] and *N. tabacum* [NC_001879]. The mVISTA program in Shuffle‐LAGAN mode (Frazer, Pachter, Poliakov, Rubin, & Dubchak, 2004) was used to perform the sequential comparison of the cp genomes with the sequence annotation information of *M. spicatum*.

### 2.4 Identification of the inversion by PCR screening and sequencing in *Myriophyllum* and close relative *Gonocarpus*

To determine the origin of the inversion observed in *M. spicatum*, its presence/absence was surveyed by PCR with primer pairs diagnostic in *Myriophyllum* (*M. spicatum, M. alterniflorum, M. aquaticum, M. dicoccum, M. heterophyllum, M. lophatum, M. oguraense, M. quitense, M. sibiricum, M. tenellum, M. ussuriense, M. variifolium, M. verrucosum, M. verticillatum*), and *Gonocarpus* (*G. micranthus*; listed in Table 1). The primer pairs were designed in either conserved *rpoB* and *trnE* or *trnC* and *trnT* protein‐coding sequences, which are flanking the inversion endpoints, to allow for the assessment of the presence or absence of the inversion. The primer pairs used were: *rpoB‐F* (5′‐CTTCCGTCAAGCCCTGATC‐3′) and *trnE‐R* (5′‐ AATCCCCGCTGCCTCCTT‐3′) as well as *trnC‐F* (5′‐CGGATTTGAACTGGGGAAAA‐3′) and *trnT‐R* (5′‐CGGATTTGAACCGATGACTTAC‐3′). Each 50 μl reaction contained 2.5 mM MgCl_2_, 0.2 mM deoxynucleoside triphosphate, 0.25 mM primers, 2.5 units of Taq polymerase, and 2–5 ng of DNA. The standard PCR amplification reactions were performed at 94°C for 2 min followed by 35 cycles of 1 min denaturation at 94°C, 1 min annealing at 55°C, 2 min extension at 72°C, and a final extension of 72°C for 7 min. PCR‐amplified DNA was purified using the QIAquick PCR purification kit and then checked on 2% agarose gels after staining with ethidium bromide. The purified products were sequenced by Sangon Biotech (Shanghai, China). Sequence assemblies and alignments followed the abovementioned methods.

### 2.5 Phylogenetic analysis

The *rpoB‐trnE* and *trnC‐trnT* inversion regions and molecular markers (ITS, *trnK*, and *matK*) used in a previous study (Moody & Les, 2010) were used for a phylogenetic analysis. Because the *rpoB‐trnE* and *trnC‐trnT* loci are absent in *L. formosana*, *P. obovata*, *P. chinense*, and *S. sarmentosum*, the *rpoB‐trnC* and *trnE‐trnT* loci were used for these species because of high homology. Alignments were performed using MAFFT version 7. 0 (Katoh & Standley, 2013) with default parameters. Three combined datasets were created: (a) *rpoB‐trnE* and *trnC‐trnT*; (b) ITS, *matK*, and *trnK*; and (c) ITS, *matK, trnK, rpoB‐trnE*, and *trnC‐trnT*. An incongruence length difference (ILD) test between the *nr*ITS and *cp*DNA was performed in PAUP v4.0b10 (Swofford, 2002) with 100 replicates, and this test indicated significant differences between data partitions (*p* < .01).

Maximum likelihood (ML), conducted using RAxML 7.0.3 (Stamatakis, 2006), and Bayesian inference (BI), conducted using MrBayes 3.1.2 (Huelsenbeck & Ronquist, 2001), were used to conduct the phylogenetic analyses. For ML analyses, values of all parameters were calculated by RAxML. Nonparametric ML bootstrap analyses included 1,000 pseudoreplicates. For BI analyses, two simultaneous runs were conducted, each consisting of four chains. In total, chains were run for 5,000,000 generations, with trees sampled every 1,000 generations. The first 25% of sampled generations were discarded as burn‐in, and the remaining trees were used to calculate majority‐rule consensus trees and posterior probabilities for nodes. Akaike information criterion (AIC) via Modeltest v3.7 (Posada & Crandall, 1998) was used to determine the most appropriate model of nucleotide evolution, supporting the use of GTR + I+G.

### 2.6 Molecular dating

Molecular dating analyses were run in BEAST package v1.7.5 (Drummond & Rambaut, 2007) using the combined ITS, *matK, trnK, rpoB‐trnE*, and *trnC‐trnT* matrix. The analysis followed the dating strategies in Chen *et al*. (Chen et al., 2014). The GTR + I + G model was selected as the best fit for the data by Mrmodeltest v2.3 (Nylander, 2004). A relaxed clock (uncorrelated lognormal) was selected as preliminary likelihood‐ratio test (LRT; Huelsenbeck & Rannala, 1997) rejected the strict molecular clock hypothesis for our data (*p* < .01). A Yule speciation model was used as a prior on the tree. We chose two reliable calibration points to constrain divergence times based on fossil taxa as follows: the extinct *Tarahumara sophiae*, representing the oldest known macrofossil record for Haloragaceae from the Maastrichtian–Campanian period (70.0 Ma) in northern Mexico (Hernandez‐Castillo & Cevallos‐Ferriz, 1999); one Altingiaceae species, *Microaltingia apocarpelata* (Zhou, Crepet, & Nixon, 2001), considered one of the oldest fossils of Saxifragales represented by macrofossils from the Upper Cretaceous (ca. 90 Ma) in New Jersey (USA). We defined 90.0 Ma as the lower boundary for the root age, and the crown group age of Haloragaceae was 70.0 Ma. Six independent Bayesian Markov chain Monte Carlo (MCMC) chains were run for 100 million generations on each, sampling every 10,000 generations. Tracer v1.5 was used to check the effective sample size (ESS) scores for all relevant estimated parameters to ensure values above 250. LogCombiner v1.7.5 was used to combine trees from these six runs and removed 25% generations as burn‐in. A maximum clade credibility tree with median ages and 95% highest posterior density (HPD) intervals was constructed using TreeAnnotator v1.7.5.

## 3 RESULTS

### 3.1 General characteristics of the *M. spicatum* cp genome

**Figure 2:**
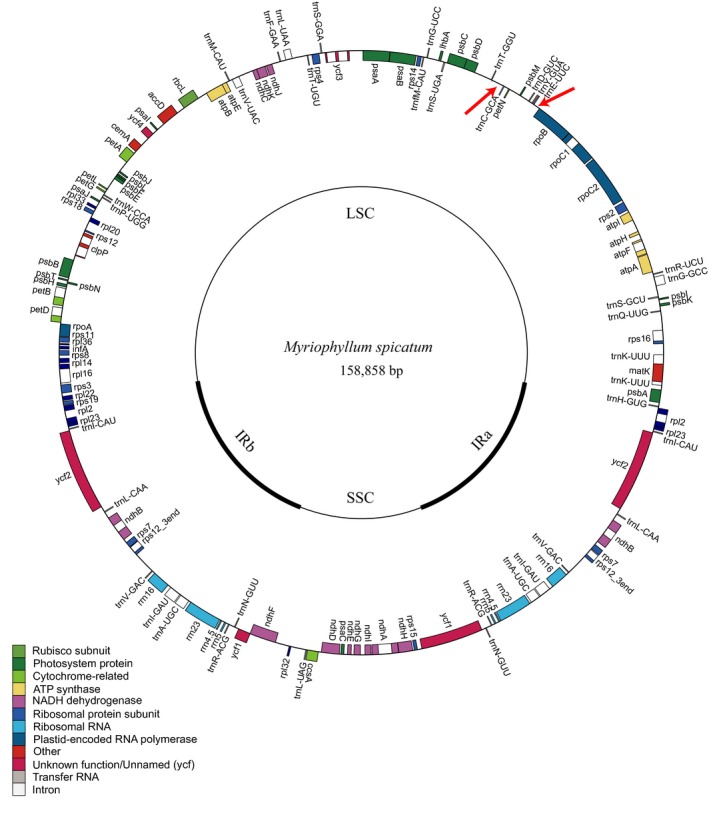
The whole assembly of the chloroplast genome of *M. spicatum*. The inverted repeats (IRa, IRb) were indicated in thick black lines on inner cycle which separate the genome into the large (LSC) and small (SSC) single copy regions. The genes drawn outside of the circle are transcribed counterclockwise, while those inside are clockwise. Gene boxes are colored by functional group as shown in the key. The red arrows denote the location of the 4‐kb inversion

The complete cp genome of *M. spicatum* (GenBank accession number: MK250869) contains 158,858 bp with a quadripartite structure, and two IRs (25,813 bp) separated by an SSC (18,814 bp) and an LSC (88,418 bp) region (Figure 2). The IR extends from *rps*19 through a portion of *ycf*1 and contains 18 duplicated genes with one or two introns. The genome contains 113 unique genes including 30 *t*RNA genes, four *r*RNA genes, and 79 protein‐coding genes (Table 2). Genes involved in photosynthesis and transcription and translation were the two dominant families. There were six genes coding the subunits of ATP synthase and 11 genes associated with the subunits of NADH dehydrogenase. The genome consists of 58% coding regions and 42% noncoding regions, including both intergenic spacers and introns. A total of 26,316 codons represent the coding capacity of 79 protein‐coding genes in the genome. The frequency of codon usage was calculated based on the sequences of protein‐coding genes and *t*RNA genes, which are summarized in Table 3. Codon usage frequency demonstrated that leucine is the most common amino acid with 2,812 codons (10.69%), while cysteine is the least common with 299 codons (1.14%).

**Table 2:** Genes present in *Myriophyllum spicatum* chloroplast genome

Category	Group of genes	Genes
Photosynthesis‐related genes (47)	Rubisco (1)	*rbcL*
	Photosystem I (5)	*psaA, psaB, psaC, psaI, psaJ*
	Assembly/stability of photosystem I (2)	ycf3**,ycf4
	Photosystem II (15)	*psbA,psbB,psbC,psbD,psbE,psbF,psbH,psbI,psbJ,psbK,psbL,psbM,psbN,psbT,psbZ*
	ATP synthase (6)	atpA, atpB, atpE, atpF*, atpH, atpI
	cytochrome b/f compelx (6)	petA, petB*, petD*, petG, petL, petN
	cytochrome c synthesis (1)	*ccsA*
	NADPH dehydrogenase (11)	ndhA*, ndhB*(x2), ndhC, ndhD, ndhE, ndhF,ndhG, ndhH, ndhI, ndhJ, ndhK
Transcription and translation‐related genes (59)	transcription (4)	rpoA, rpoB, rpoC1*, rpoC2
	ribosomal proteins (20)	rps2, rps3, rps4, rps7(x2), rps8, rps11, rps12*(x2), rps14,rps15, rps16*, rps18, rps19,rpl2*(x2), rpl14, rpl16*, rpl20, rpl23(x2), rpl32, rpl33,rpl36
	translation initiation factor (1)	*infA*
	ribosomal RNA (4)	*rrn5(x2), rrn4.5(x2), rrn16(x2), rrn23(x2)*
	transfer RNA (30)	trnA‐UGC*(x2), trnC‐GCA, trnD‐GUC, trnE‐UUC, trnF‐GAA,,trnG‐UCC,trnG‐GCC*, trnH‐GUG, trnI‐CAU(x2), trnI‐GAU*(x2),trnK‐UUU*, trnL‐CAA(x2), trnL‐UAA*, trnL‐UAG, trnfM‐CAUI,trnM‐CAU, trnN‐GUU(x2), trnP‐UGG, trnQ‐UUG,trnR‐ACG(x2), trnR‐UCU, trnS‐GCU, trnS‐GGA, trnS‐UGA, trnT‐GGU,trnT‐UGU, trnV‐GAC(x2), trnV‐UAC*, trnW‐CCA, trnY‐GUA
Other genes (6)	RNA processing (1)	*matK*
	carbon metabolism (1)	*cemA*
	fatty acid synthesis (1)	*accD*
	proteolysis (1)	clpP**
	conserved genes with unknown functions (2)	*ycf1, ycf2(x2), ycf15(x2)*

Note

One and two superscript asterisks indicate one‐ and two‐intron‐containing genes, respectively. Genes located in the IR region are indicated by (x2) after the gene name.

**Table 3:** Codon usage in *Myriophyllum spicatum* chloroplast genome

Codon	Amino acid	Count	RSCU	tRNA	Codon	Amino acid	Count	RSCU	tRNA
UUU(F)	Phe (F)	988	1.31		UCU(S)	Ser (S)	564	1.68	
UUC(F)	Phe (F)	524	0.69	trnF‐GAA	UCC(S)	Ser (S)	304	0.90	trnS‐GGA
UUA(L)	Leu (L)	870	1.86	trnL‐UAA	UCA(S)	Ser (S)	409	1.22	trnS‐UGA
UUG(L)	Leu (L)	560	1.19	trnL‐CAA	UCG(S)	Ser (S)	198	0.59	
CUU(L)	Leu (L)	593	1.27		CCU(P)	Pro (P)	424	1.58	
CUC(L)	Leu (L)	199	0.42		CCC(P)	Pro (P)	202	0.75	trnP‐UGG
CUA(L)	Leu (L)	394	0.84	trnL‐UAG	CCA(P)	Pro (P)	315	1.17	
CUG(L)	Leu (L)	196	0.42		CCG(P)	Pro (P)	135	0.50	
AUU(I)	I le (I)	1,103	1.45		ACU(T)	Thr (T)	542	1.61	
AUC(I)	I le (I)	418	0.55	trnI‐GAU	ACC(T)	Thr (T)	243	0.72	trnT‐GGU
AUA(I)	I le (I)	748	1.12	trnI‐CAU	ACA(T)	Thr (T)	407	1.21	trnT‐UGU
AUG(M)	Met (M)	590	0.88	trnM‐CAU	ACG(T)	Thr (T)	156	0.46	
GUU(V)	Val (V)	520	1.48		GCU(A)	Ala (A)	637	1.82	
GUC(V)	Val (V)	175	0.50	trnV‐GAC	GCC(A)	Ala (A)	230	0.66	
GUA(V)	Val (V)	525	1.49	trnV‐UAC	GCA(A)	Ala (A)	401	1.15	trnA‐UGC
GUG(V)	Val (V)	186	0.53		GCG(A)	Ala (A)	129	0.37	
UAU(Y)	Try (Y)	785	1.62		UGU(C)	Cys (C)	227	1.52	
UAC(Y)	Try (Y)	185	0.38	trnY‐GUA	UGC(C)	Cys (C)	72	0.48	
UAA(*)	Stop	49	0.27		UGA(*)	Stop	15	0.06	trnS‐GCU
UAG(*)	Stop	21	0.12		UGG(W)	Trp (W)	460	1.94	trnC‐GCA
CAU(H)	His (H)	471	1.52		CGU(R)	Arg (R)	340	1.48	
CAC(H)	His (H)	150	0.48		CGC(R)	Arg (R)	99	0.43	trnW‐CCA
CAA(Q)	Gln (Q)	712	1.51	trnH‐GUG	CGA(R)	Arg (R)	354	1.54	trnR‐ACG
CAG(Q)	Gln (Q)	231	0.49	trnQ‐UUG	CGG(R)	Arg (R)	129	0.56	
AAU(N)	Asn (N)	968	1.52		AGU(S)	Ser (S)	446	1.33	
AAC(N)	Asn (N)	305	0.48		AGC(S)	Ser (S)	95	0.28	
AAA(K)	Lys (K)	1,080	1.51	trnN‐GUU	AGA(R)	Arg (R)	500	2.76	trnR‐UCU
AAG(K)	Lys (K)	350	0.49	trnK‐UUU	AGG(R)	Arg (R)	154	0.85	
GAU(D)	Asp (D)	887	1.63		GGU(G)	Gly (G)	595	1.32	
GAC(D)	Asp (D)	201	0.37		GGC(G)	Gly (G)	168	0.37	trnG‐GCC
GAA(E)	Glu (E)	1,007	1.50	trnD‐GUC	GGA(G)	Gly (G)	716	1.59	trnG‐UCC
GAG(E)	Glu (E)	338	0.50	trnE‐UUC	GGG(G)	Gly (G)	321	0.71	

Note

Excluding pseudogenes.

### 3.2 Repeat analysis

**Figure 3:**
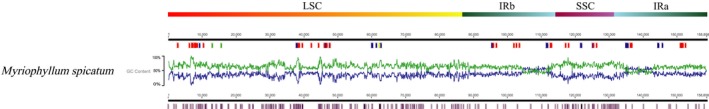
Distribution of repeat sequences and SSRs in *M. spicatum* chloroplast genome. GC content is shown

A total of 38 repeats were found including 21 direct (forward) repeats, 15 inverted (palindrome) repeats, one reverse repeat, and one complement repeat (Table S1). The longest repeat is a 51‐bp inverted repeat between the *rbcL* and *accD*. Most of the repeats are distributed within the intergenic spacer regions, the intron sequences, and *ycf1* and *ycf2*. Cp microsatellites (*cp*SSRs) are potentially useful markers for detection of polymorphisms (Provan, Powell, & Hollingsworth, 2001); therefore, the distribution of SSRs was also analyzed, and 260 SSRs were identified in total. Among the identified SSRs, 177 mononucleotide SSRs (68.08%), 66 dinucleotide SSRs (25.38%), seven trinucleotide SSRs (2.70%), and 10 tetranucleotide SSRs (3.84%) were recognized. Most homopolymers are constituted by A/T sequences (98.87%). Of the dipolymers, 75.76% were constituted by multiple A and T bases. One hundred and fifty‐nine of the SSR loci were found in the intergenic regions, 35 were located in introns, and the other 66 SSRs were located in genes (Table S2). The locations of repeat sequences and SSRs are shown in Figure 3.

### 3.3 Comparison of genome organization in Saxifragales

**Figure 4:**
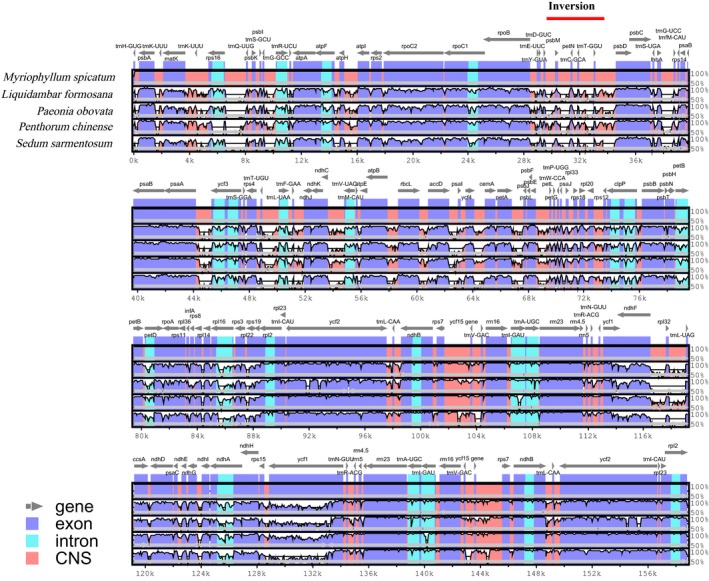
Comparison of five Saxifragales chloroplast genome. The top gray arrows and thick black lines show genes with their orientation. The inversion was indicated by thick red line. The y‐axis represents the percent identity within 50%–100%. The *x*‐axis represents the coordinate in the cp genome. Genome regions are color‐coded as protein‐coding (exon), intron, and conserved noncoding sequences (CNS)

To understand the structural characteristics in the cp genomes of *M. spicatum*, *L. formosana*, *P. obovata*, *P. chinense*, and *S. sarmentosum*, and broadly, Saxifragales, the size, gene content, and organization of the cp genomes were sampled for comparative analysis. The characters of the genomes from the abovementioned species are listed in Table S3. As expected, there were considerable differences in terms of genome size, GC content, extent of IR, gene content, and gene order. The coding region in *M. spicatum* was the largest (92,088 bp) among the five Saxifragales species investigated. The GC content of the LSC region, SSC region, and IRs of *M. spicatum* was the lowest. To understand the structural characteristics in the cp genome of *M. spicatum*, the comparative sequence alignment of the cp genome sequences of the five Saxifragales species was performed with the new annotation of *M. spicatum* as a reference (Figure 4). This showed general conservativeness among the five species but with some highly varied regions, including *ycf1, rps16, ndhA*, and *accD*, occurring as the most divergent coding genes.

**Figure 5:**
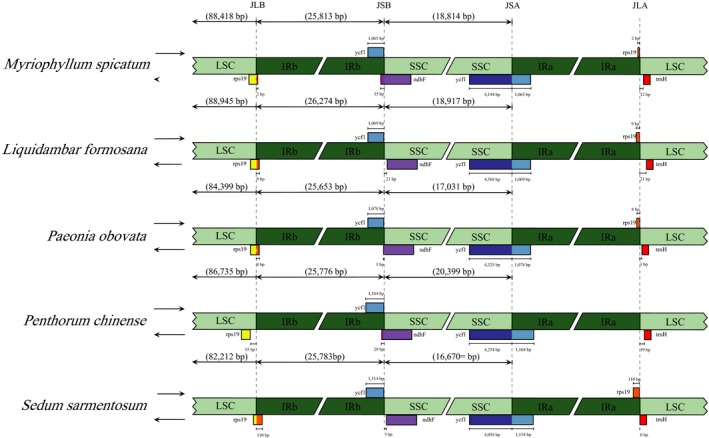
Comparison of the borders of LSC, IR, SSC, and LSC regions among five Saxifragales genomes. The adjacent border genes are indicated by boxes with gene names and bps above or below the main line

The exact borders between the IR regions and the two single copy regions (LSC and SSC) were also compared to investigate the contraction or expansion of the IR regions (Figure 5). We found that the IR/SSC boundary regions were slightly varied. The genes marking the beginning and end of the IR were only partially duplicated. Specifically, 2–110 bp of *rps19* (except for in *P. chinense*, which was entirely located in the LSC) and 1,065–1,164 bp of *ycf1*. The *rps19* pseudogene occurred at the end of IRa and the *ycf1* pseudogene occurred at the end of IRb. The *ndhF* gene shares some nucleotides with the *ycf1* pseudogene (35 bp in *M. spicatum*, 1 bp in *P. obovata*, and 29 bp in *P. chinense*). Neither gene loss nor intron loss were detected in the cp genome of *M. spicatum*. *ycf15* is identified as a pseudogene in the cp genome of *M. spicatum* because of the presence of a premature stop codon, which is different from the other four Saxifragales species.

### 3.4 Occurrence of the unique lineage‐specific inversion

**Figure 6:**
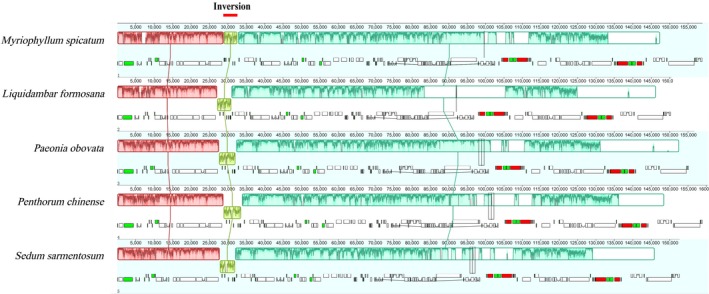
Linearized maps comparison of the plastid genomes of five Saxifragales plants. Syntenic blocks are shown above and gene maps are shown below. Unique regions are boxed in yellow, and the inversion events occurred in *M. spicatum* are marked with short red line

A 4‐kb inverted fragment in the LSC between *rpoB‐trnT* was found in *M. spicatum* after comparison with the four other taxa from Saxifragales (Figure 6). One end point of the inversion is located between the *rpoB* and *trnE* and ~300 bp upstream to *trnE*. The other end point is located between the *trnC‐UUC* and *trnT‐GGU*, ~1,000 bp downstream to *trnC*. The break points do not disrupt any genes. The *trnE‐trnC* inversion contained four *t*RNA genes (*trnE, trnY, trnD*, and *trnC*) and two protein‐coding genes (*psbM* and *petN*). To verify the presence of the inversion in *Myriophyllum*, we investigated 13 other *Myriophyllum* species (*M. alterniflorum, M. aquaticum, M. dicoccum, M. heterophyllum, M. lophatum, M. oguraense, M. quitense, M. sibiricum, M. tenellum, M. ussuriense, M. variifolium, M. verrucosum, M. verticillatum*) as well as *G. micranthus* in Haloragaceae, which is a species in a closely related genus. PCR amplification guided by four designed primers confirmed the presence the 4‐kb inversion among all of these species. Moreover, *L. formosana*, *P. obovat*a, *P. chinense*, and *S. sarmentosum* lacked this 4‐kb inversion.

**Figure 7:**
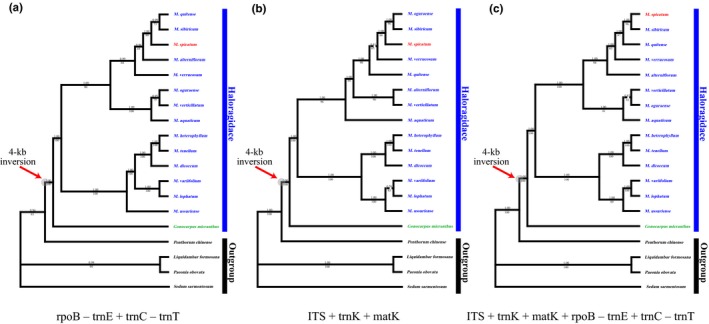
Inferred phylogenetic trees of 15 taxa of Haloragidaceae and related families basing on maximum (ML) and Bayesian inference (BI) analyses of different combined datasets. (a) rpoB‐trnE+trnC‐trnT. (b) ITS+trnK+matK. (c) ITS+trnK+matK+rpoB‐trnE+trnC‐trnT. The ML bootstrap values (below) and Bayesian posterior probability (above) are given for each branch. The 4‐kb inversion rearrangement event was mapped onto the branches with red arrow

**Figure 8:**
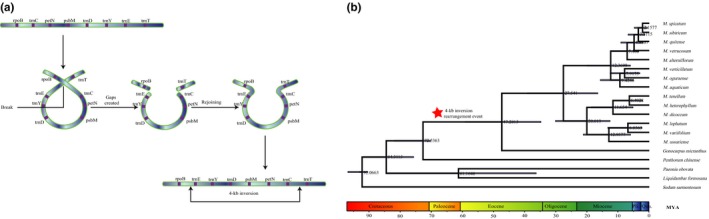
(a) Illustration of the suggested flip‐flop recombination event in Haloragidaceae resulting in a 4‐kb inversion. The ribbons represent partial of the chloroplast genome, and the genes are colored in purple. (b) Chronogram of Haloragaceae predicts and estimates the origin of the 4‐kb inversion under a Bayesian relaxed clock model by using of the combined ITS, *matK*, *trnK*, *rpoB‐trnE*, and *trnC‐trnT* matrix. Gray colored bars at nodes indicate the 95% credibility intervals of age estimates. The numbers near the nodes refer to the node age. Red asterisks highlight the 4‐kb inversion rearrangement event

The phylogenetic relationships among 15 Haloragaceae species and four outgroup species (*L. formosana*, *P. obovat*a, *P. chinense*, and *S. sarmentosum*) were investigated using three combined datasets (Figure 7). Two highly supported with strong bootstrap value (100%) monophyletic groups were identified within the 14 *Myriophyllum* species (Figure 6). *Gonocarpus micranthus* clustered into *Myriophyllum*, indicating that additional detailed analyses are needed including more species of *Myriophyllum*, *Gonocarpus*, and other closely related genera. The results also showed that *P. chinense* was more closely related to Haloragaceae rather than other outgroup species. Our results are congruent with the previous phylogenetic analysis among families of Saxifragales (Dong, Xu, Cheng, Lin, et al., 2013; Dong, Xu, Cheng, & Zhou, 2013; Jian et al., 2008; Moody & Les, 2010). The 4‐kb inversion originated after the split of Haloragaceae and Penthoraceae but before the divergence of *Myriophyllum* and *Gonocarpus*; the 4‐kb inversion was identified in all of the included *Myriophyllum* species and the *Gonocarpus* taxa. Bayesian analysis in BEAST and molecular dating (Figure 8b) further suggested that the *trnE‐trnC* inversion might have occurred between upper Cretaceous (72.54 MYA) and middle Eocene (47.28 MYA).

## 4 DISCUSSION

In this study, the complete cp genome of *M. spicatum* was assembled, and it possesses the typical angiosperm quadripartite structure with two short inverted repeat regions separated by two single copy regions. The length of the IR regions of *M. spicatum* is similar to that of other Saxifragales species, and the genome size of the cp genome of the Saxifragales species investigated here only varied slightly, ranging from 152,698 to 160,410 bp. These results suggest that genomic length variation can be found in the LSC and SSC boundary regions, as reported for other species (Zhao et al., 2018). Only the *ycf1* pseudogene was detected across the SSC/IRa border in the five Saxifragales species, which might be caused by a duplication of the normally single copy gene *ycf*1. No stop codons were detected in the coding sequence of *ycf*1; thus, we hypothesize that the expansion of the IR was caused by a duplication of *ycf*1, which occurred in the common ancestor of these species in Saxifragales.

Based upon the alignment of the plastomes from five Saxifragales species, the gene contents were almost identical. Most variations were detected in intergenic regions yet are also highly variable in coding regions such as *ycf*1, *rps*16, *ndhA*, and *accD*. These highly variable regions may be useful as specific DNA barcodes for species‐level identification, as well as provide genetic markers for resolving relationships among Saxifragales. Over 260 SSRs were identified in this study, which could be candidates for future inferences on population genetics and help to trace the origin of invasive populations (Provan et al., 2001). Moreover, these SSR markers could be used for genetic diversity studies on closely related species in Haloragaceae.

Normally, plastomic rearrangements in flowering plants are rare (Mower et al., 2018). Most photosynthetic angiosperms have a highly conserved plastome organization, except a small number of groups among major lineages, especially the Campanulaceae, Fabaceae, and Geraniaceae, which exhibit remarkable and extensive rearrangements (Jansen & Ruhlman, 2012; Mower & Vickrey, 2018)). In this article, a 4‐kb inversion was identified in all *Myriophyllum* species sampled and therefore likely provides an informative marker that highlights an additional synapomorphy supporting the monophyly of *Myriophyllum*. Moreover, the activity of repetitive elements has often been considered to be associated with plastome rearrangement and recombination (Lu et al., 2017; Weng, Blazier, Govindu, & Jansen, 2013). Regarding the *trn*E‐*trn*C inversion in *Myriophyllum*, a flip‐flop recombination event might have contributed to its occurrence (Figure 8a). This detectable rearrangement of sequences has occurred during the evolution of *Myriophyllum*, possibly playing an important role in the maintenance of the structural stability of the chloroplast genome (Palmer & Thompson, 1982; Wolfe, Li, & Sharp, 1987).

The 4‐kb inversion was detected in *G. micranthus*, a species in a genus closely related to *Myriophyllum* (Chen et al., 2014). Our results are congruent with the previous phylogenetic analysis among families of Saxifragales (Jian et al., 2008; Moody & Les, 2010; Dong, Xu, Cheng, Lin, et al., 2013; Dong, Xu, Cheng, & Zhou, 2013). The 4‐kb inversion was identified in all of the included *Myriophyllum* species and the *Gonocarpus* taxa; thus, the 4‐kb inversion might originate after the split of Haloragaceae and Penthoraceae but before the divergence of *Myriophyllum* and *Gonocarpus*. The molecular dating calibrated by fossil records indicated that *Myriophyllum* and *Gonocarpus* separated approximately 47 MYA, and the *Myriophyllum‐Gonocarpus* clade diverged approximately 72 MYA in Saxifragales. Based on historical biogeography analysis of Haloragaceae (Chen et al., 2014), our study indicated that this 4‐kb inversion was likely shared by a majority of clades in Haloragaceae (including almost three quarters of the species in Haloragaceae) before the earliest diversification of this family. A clustering of angiosperm paleopolyploidizations occurred around the Cretaceous–Paleogene (K–Pg) extinction event about 66 million years ago based on dated genome data (Vanneste, Baele, Maere, & Yves, 2014). Thus, we speculate that the 4‐kb inversion might be caused by an occurrence of nonrandom recombination associated with climate changes around the K–Pg boundary (Kaiho et al., 2016; Vellekoop et al., 2015). Additional whole chloroplast genome sequences from species in Haloragaceae should be obtained to construct larger phylogenetic trees to further test this presumption. In addition, more functional investigations are also needed to provide a more comprehensive understanding of divergence history and the influence of climate change on the novel 4‐kb inversion.

## CONFLICT OF INTEREST

The authors declare no conflict interest.

## AUTHOR CONTRIBUTIONS

TW and FL designed the study and modified manuscript. FL and YYL conducted the sequence analyses and drafted the manuscript. YL and RWM performed the experiments and analyzed the data. XL and TFL collected the samples. All authors read and approved the final manuscript.

## Data Availability

The complete chloroplast genome of *Myriophyllum spicatum* has been deposited in GenBank (Accession Number: MK250869).
